# Association between serum potassium levels and hearing loss: Evidence from a cross-sectional study based on NHANES

**DOI:** 10.1097/MD.0000000000049873

**Published:** 2026-07-24

**Authors:** Xiao-Zou Luo, Jian Luo, Jie Shen, Deng-Chao Wang

**Affiliations:** a Department of Otorhinolaryngology Head and Neck Surgery, The First People’s Hospital of Yibin, Yibin, Sichuan, China; b Department of General Surgery, Zigong Fourth People’s Hospital, Zigong, Sichuan, China.

**Keywords:** hearing loss, NHANES, serum potassium

## Abstract

Hearing loss is a global public health issue, and potassium imbalance may affect inner ear function, thereby impacting hearing. Currently, research on the association between serum potassium levels and hearing loss remains limited. This study utilized National Health and Nutrition Examination Survey data to investigate the relationship between serum potassium levels and hearing loss, offering a theoretical basis for the prevention of hearing loss. National Health and Nutrition Examination Survey data were analyzed using multivariable logistic regression models to examine the association between serum potassium levels and hearing loss. To explore potential nonlinear relationships, restricted cubic spline analysis was performed. Subgroup analyses and interaction tests were also carried out to investigate how this association varied across different populations. The study included 10,009 eligible participants, among whom 2952 (29.5%) were diagnosed with speech-frequency hearing loss (SFHL), 2216 (22.1%) with low-frequency hearing loss (LFHL), and 4688 (46.8%) with high-frequency hearing loss (HFHL). The results indicated a significant positive association between serum potassium levels and SFHL, LFHL, and HFHL (SFHL: odds ratio [OR] = 1.49, 95% confidence interval [CI]: 1.15–1.94, *P* = .003; LFHL: OR = 1.45, 95% CI: 1.16–1.82, *P* = .002; HFHL: OR = 1.57, 95% CI: 1.29–1.92, *P* < .001). Trend tests showed *P* < .05. Restricted cubic spline analysis demonstrated a linear relationship between serum potassium levels and SFHL, LFHL, and HFHL. Interaction tests suggested that factors such as sex, race/ethnicity, and diabetes status significantly modified the association between serum potassium levels and hearing loss. Elevated serum potassium levels are significantly positively associated with the prevalence of hearing loss. These findings provide new insights into the potential role of serum potassium in auditory health and may offer strategies for early prevention and metabolic intervention in hearing loss.

## 1. Introduction

Hearing loss is currently a globally recognized public health issue, particularly prevalent among the elderly. Its incidence rises significantly with age. Hearing loss not only diminishes quality of life but also increases the risk of cognitive decline and depression to some extent.^[1,2]^ The causes of hearing loss are multifactorial, encompassing a wide range of endogenous and exogenous factors, including aging-related degeneration, vascular risk factors, ototoxic exposures, noise-induced trauma, inner-ear disorders, and genetic susceptibility.^[3–7]^ In addition, tinnitus is a common consequence of auditory dysfunction and is closely associated with hearing impairment, often co-occurring with hearing loss and further diminishing quality of life.^[8]^ Given the growing global burden, understanding the underlying mechanisms and influencing factors of hearing loss is crucial for effective prevention and treatment.

Potassium ions are the most abundant cations inside cells and play a key role in maintaining the normal function of the auditory system. They are crucial in the inner ear’s signal transmission process. Potassium ion channels in the inner ear precisely regulate potassium ion transport, maintaining the electrochemical balance of endolymph and hair cells, thus ensuring proper signal transmission.^[9]^ Dysfunction of specific potassium ion channels, such as potassium voltage-gated channel subfamily Q member 4 (KCNQ4) and big potassium channels, can lead to progressive hearing loss.^[10]^ Additionally, aquaporin-4 in supporting cells helps maintain the balance of water and potassium ions, thereby supporting the normal function of the inner ear.^[11]^ These findings underscore the importance of potassium homeostasis in auditory health, with disturbances in potassium homeostasis potentially contributing to auditory damage.

This study aims to analyze National Health and Nutrition Examination Survey (NHANES) data to explore the potential association between serum potassium levels and hearing loss. By analyzing serum potassium data and hearing loss indicators in this dataset, we hope to reveal the connection between serum potassium levels and hearing loss, while controlling for potential confounding factors. The findings are expected to deepen our understanding of the role of serum potassium levels in auditory health and provide a scientific basis for early detection and risk prediction of hearing loss.

## 2. Materials and methods

### 2.1. Data source

This research utilized data from the NHANES database spanning multiple cycles between 2005 to 2012 and 2015 to 2018. NHANES is a national survey managed by the Centers for Disease Control and Prevention in the United States, which assesses the health and nutritional status of a randomly selected sample of the U.S. population. The database provides extensive data on diseases, nutrition, and metabolic indicators. Data collection was conducted through interviews, physical examinations, and laboratory tests, ensuring comprehensive and reliable research data, and offering diverse research variables and background information for this study. We extracted variables related to serum potassium levels and hearing, and included covariates such as age, sex, race, body mass index (BMI), and chronic diseases (e.g., diabetes and hypertension) to control for potential confounding factors and analyze their impact on hearing health. All data in the NHANES database are publicly accessible and do not contain personally identifiable information, thus no additional ethical approval was required.^[12,13]^

### 2.2. Study population

Data from 6 NHANES survey cycles (2005–2012 and 2015–2018) were analyzed. To ensure that the study population represented the adult population, individuals younger than 20 years were excluded. Participants with missing data on serum potassium levels or hearing loss were also excluded.

### 2.3. Definition of hearing loss

In this study, the assessment of hearing loss was based on the standard pure-tone air-conduction audiometry method used in NHANES. Before pure-tone audiometry, participants underwent a brief otoscopic examination and tympanometry as part of the standardized NHANES audiological protocol to identify external or middle ear conditions that could contribute to conductive hearing loss. Testing was conducted by highly trained examiners in calibrated soundproof rooms using standard and insert earphones to ensure accuracy and reliability. Participants underwent bilateral hearing tests at 7 different frequencies (0.5, 1, 2, 3, 4, 6, and 8 kHz), with intensity levels ranging from −10 to 120 decibels (dB). To ensure consistency and reliability of the data, the 1 kHz frequency was tested twice in each ear, and results with a discrepancy >10 dB were excluded. Detailed descriptions of the measurement methods can be found in the National Center for Health Statistics Hearing Examination Procedures Manual. The speech-frequency pure-tone average was calculated as the mean hearing threshold at 0.5, 1, 2, and 4 kHz and was used to define speech-frequency hearing loss (SFHL). The calculation of the low-frequency pure-tone average is based on the average hearing threshold levels at the frequencies of 0.5, 1, and 2 kHz, while the high-frequency pure-tone average is derived from the data at 3, 4, and 6 kHz. In the study, the a pure-tone average (PTA) results for both ears of each participant were included in the comparison, with the ear showing better hearing ultimately being used as the primary basis for assessing hearing. According to the World Health Organization standards outlined in the “World Report on Hearing,” a PTA of ≥20 dB is considered indicative of hearing loss. Based on this, if the values of speech-frequency pure-tone average, low-frequency pure-tone average, and high-frequency pure-tone average of ≥20 dB, they are defined as SFHL, low-frequency hearing loss (LFHL), and high-frequency hearing loss (HFHL), respectively.^[14,15]^

### 2.4. Measurement of serum potassium

The data on serum potassium levels were extracted from the NHANES database, which was obtained using standardized laboratory methods. Blood samples were collected as serum specimens by trained professionals using BD Vacutainer red-top tubes (Becton, Dickinson and Company, Franklin Lakes; without anticoagulant) in accordance with standardized NHANES venipuncture protocols. Blood specimens were processed under strict quality control to ensure accuracy and consistency, and serum was separated by centrifugation and stored under frozen conditions prior to analysis, following NHANES laboratory documentation. Potassium ion concentrations in the serum were measured using the ion-selective electrode method. According to commonly accepted clinical standards, normal serum potassium levels in adults typically range from 3.5 to 5.0 mmol/L. All serum potassium measurements underwent quality checks, with invalid and missing values excluded to minimize potential measurement errors and bias. The results were reported in millimoles per liter (mmol/L).

### 2.5. Covariates

This study incorporated a range of covariates to control for potential confounders influencing the association between serum potassium levels and hearing loss. Demographic variables included age (20–39, 40–59, and ≥60 years), sex (male or female), race/ethnicity (Mexican American, other Hispanic, non-Hispanic White, non-Hispanic Black, and other races), education level (less than high school, high school or equivalent, and college or above),^[16]^ and marital status (married, widowed, divorced, separated, never married, or living with a partner). Economic status was assessed using the poverty income ratio (PIR). Health and lifestyle factors included daily potassium and alcohol intake, hypertension (binary: yes/no), high cholesterol levels (binary: yes/no), diabetes (binary: yes/no), and BMI. Smoking status was categorized as never smoked, former smoker, and current smoker.^[17]^ Sleep disorders and depressive symptoms were recorded as binary variables (yes/no). Environmental and biochemical variables included noise exposure, assessed by whether participants had been exposed to firearm or other loud noises, and measurements of serum creatinine, total calcium, and sodium levels. The inclusion of these covariates aimed to minimize confounding effects, ensuring a more accurate understanding of the association between serum potassium levels and hearing loss.

### 2.6. Statistical analyses

Serum potassium levels were categorized into 4 quartiles (*Q*1–*Q*4). The normality of continuous variables was assessed using the skewness–kurtosis test. For handling missing values, categorical variables were imputed using the mode, and continuous variables with a skewed distribution were imputed with the median. Categorical variables were presented as percentages and compared using the weighted chi-square test. Continuous variables that were not normally distributed were expressed as median and interquartile range and compared across groups using the weighted Kruskal–Wallis test. To examine the association between serum potassium levels and the prevalence of hearing loss, 3 multivariable logistic regression models were constructed: model 1: unadjusted model; model 2: adjusted for age, sex, race/ethnicity, education level, marital status, and PIR; model 3: additionally adjusted for hypertension, high cholesterol, diabetes, depression, sleep disorder, BMI, serum creatinine, serum total calcium, serum sodium, daily potassium intake, daily alcohol intake, smoking status, and exposure to firearm or loud noise. Odds ratios (ORs) and 95% confidence intervals (CIs) were calculated in all models to assess the strength of association. Restricted cubic spline (RCS) analysis was performed to explore potential nonlinear relationships between serum potassium levels and hearing loss. Subgroup analyses were conducted to evaluate differences in this association among various populations, and interaction tests were performed to assess potential effect modification by covariates. All statistical analyses were performed using R software (version 4.2.2; R Foundation for Statistical Computing) and Free Statistics software (version 1.9; Beijing Fengrui Colin Medical Technology Co., Ltd., Beijing, China). A 2-sided *P* value <.05 was considered statistically significant.

## 3. Results

### 3.1. Study population selection

**Figure 1.**
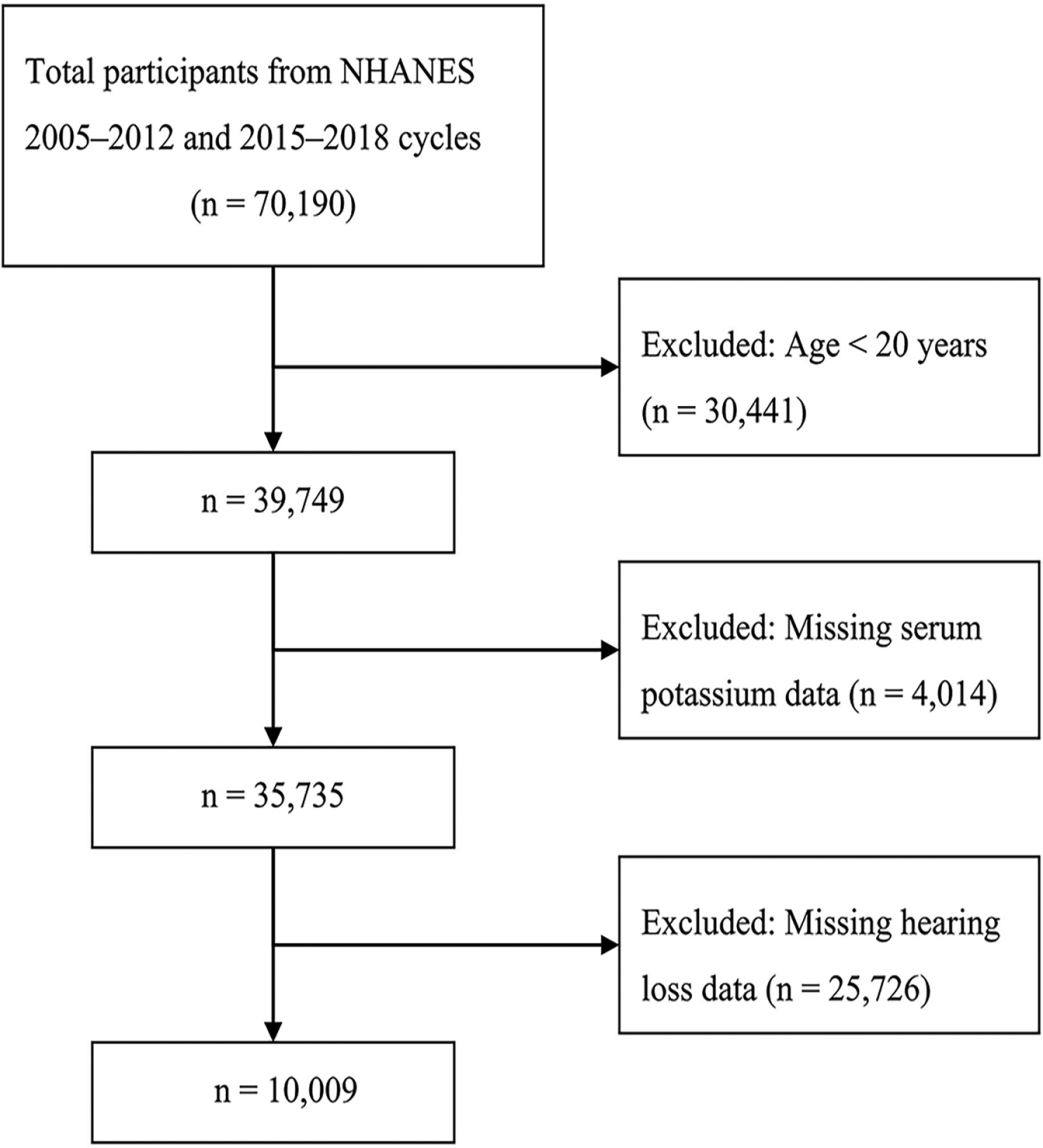
Flow diagram of the study design. NHANES = National Health and Nutrition Examination Survey.

A total of 70,190 participants from NHANES 2005 to 2012 and 2015 to 2018 cycles were considered. After excluding individuals aged <20 years (n = 30,441), those with missing serum potassium data (n = 4014), and those with missing hearing loss data (n = 25,726), 10,009 participants were included in the final analysis (Fig. 1).

### 3.2. Participants and demographic characteristics

**Figure 2.**
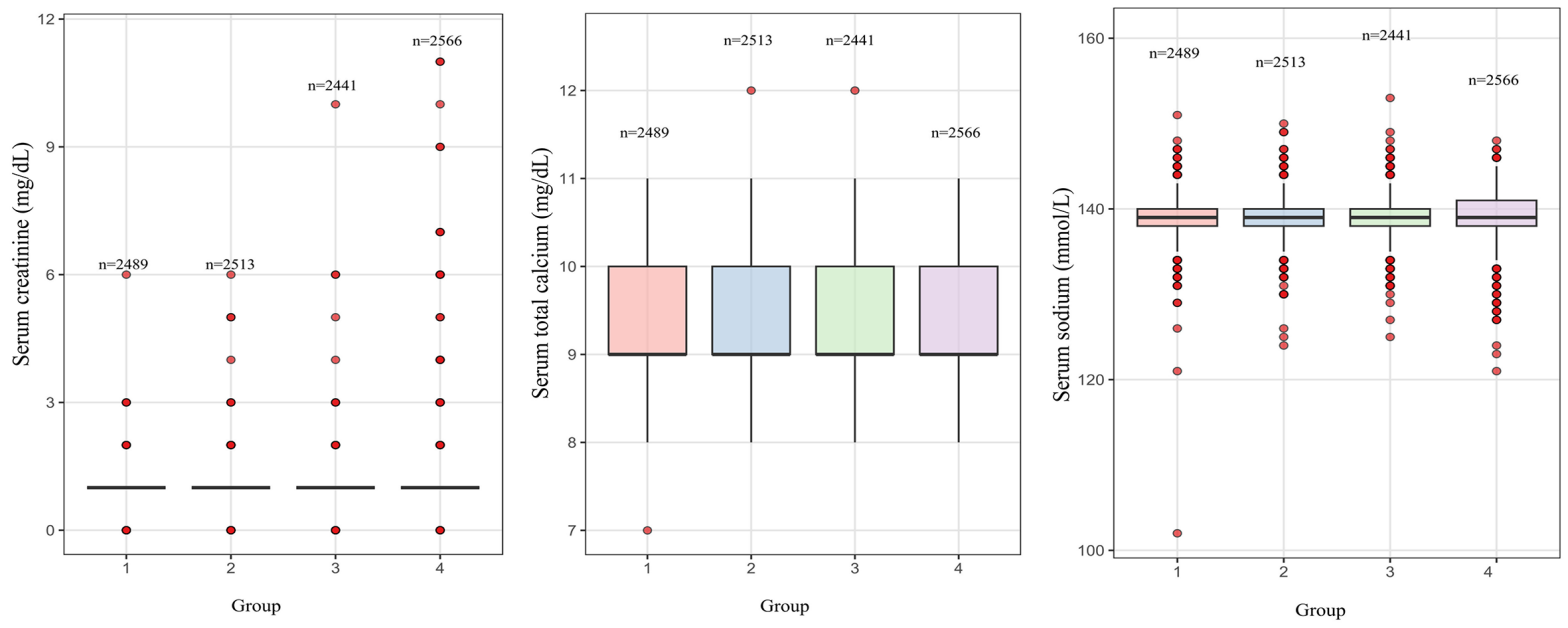
Box plots illustrating serum creatinine, serum total calcium, and serum sodium levels across the 4 potassium quartiles (*Q*1–*Q*4). The boxes represent the interquartile range, with the median indicated by the horizontal line within each box. Red dots represent outliers. Participants were divided into 4 groups based on serum potassium levels: *Q*1 (2.40–3.72 mmol/L), *Q*2 (3.73–3.93 mmol/L), *Q*3 (3.94–4.19 mmol/L), and *Q*4 (4.20–6.60 mmol/L).

Among the 10,009 participants, 4980 (49.8%) were male and 5029 (50.2%) were female. Regarding age distribution, 3113 participants (31.1%) were aged 20 to 39 years, 3040 (30.4%) were aged 40 to 59 years, and 3856 (38.5%) were aged 60 years or older. Overall, 2952 participants (29.5%) had SFHL, 2216 (22.1%) had LFHL, and 4688 (46.8%) had HFHL. Participants were divided into 4 groups according to serum potassium levels: *Q*1 (2.40–3.72 mmol/L), *Q*2 (3.73–3.93 mmol/L), *Q*3 (3.94–4.19 mmol/L), and *Q*4 (4.20–6.60 mmol/L). Significant differences were observed across potassium quartiles in SFHL, LFHL, HFHL, as well as in age, sex, race/ethnicity, education level, marital status, PIR, hypertension, high cholesterol, diabetes, BMI, serum creatinine, serum total calcium, serum sodium, daily potassium intake, daily alcohol intake, smoking status, and exposure to firearm or loud noise (*P* < .05 for all comparisons). Boxplots showing serum creatinine, serum total calcium, and serum sodium levels across the potassium quartiles are presented in Figure 2. Participants in the *Q*4 group were more likely to have SFHL, LFHL, and HFHL. They were predominantly aged ≥60 years, male, and non-Hispanic White, and tended to have lower educational attainment. In addition, the prevalence of hypertension, high cholesterol, and diabetes was higher in this group. *Q*4 participants also showed higher serum creatinine and sodium levels, greater potassium intake, a higher proportion of former smokers, and more frequent exposure to firearm and loud noise environments (Table 1).

**Table 1:** Baseline characteristics of the study participants.

		Serum potassium quartiles (mmol/L)				
		*Q*1 (2.4–3.72)	*Q*2 (3.73–3.93)	*Q*3 (3.94–4.19)	*Q*4 (4.2–6.6)	
Variables	Total (n = 10,009)	(n = 2489)	(n = 2513)	(n = 2441)	(n = 2566)	*P* value
SFHL, n (%)						<.001
No	7057 (70.5)	1982 (79.6)	1949 (77.6)	1759 (72.1)	1367 (53.3)	
Yes	2952 (29.5)	507 (20.4)	564 (22.4)	682 (27.9)	1199 (46.7)	
LFHL, n (%)						<.001
No	7793 (77.9)	2097 (84.3)	2101 (83.6)	1939 (79.4)	1656 (64.5)	
Yes	2216 (22.1)	392 (15.7)	412 (16.4)	502 (20.6)	910 (35.5)	
HFHL, n (%)						<.001
No	5321 (53.2)	1589 (63.8)	1506 (59.9)	1338 (54.8)	888 (34.6)	
Yes	4688 (46.8)	900 (36.2)	1007 (40.1)	1103 (45.2)	1678 (65.4)	
Age, n (%)						<.001
20–9 yr	3113 (31.1)	946 (38)	947 (37.7)	774 (31.7)	446 (17.4)	
40–59 yr	3040 (30.4)	825 (33.1)	805 (32)	812 (33.3)	598 (23.3)	
≥60 yr	3856 (38.5)	718 (28.8)	761 (30.3)	855 (35)	1522 (59.3)	
Sex, n (%)						<.001
Male	4980 (49.8)	990 (39.8)	1177 (46.8)	1280 (52.4)	1533 (59.7)	
Female	5029 (50.2)	1499 (60.2)	1336 (53.2)	1161 (47.6)	1033 (40.3)	
Race/ethnicity, n (%)						<.001
Mexican American	1345 (13.4)	385 (15.5)	387 (15.4)	336 (13.8)	237 (9.2)	
Other Hispanic	1007 (10.1)	244 (9.8)	280 (11.1)	259 (10.6)	224 (8.7)	
Non-Hispanic White	4087 (40.8)	789 (31.7)	878 (34.9)	1050 (43)	1370 (53.4)	
Non-Hispanic Black	2166 (21.6)	618 (24.8)	573 (22.8)	493 (20.2)	482 (18.8)	
Other race	1404 (14.0)	453 (18.2)	395 (15.7)	303 (12.4)	253 (9.9)	
Education level, n (%)						<.001
Less than high school	2283 (22.8)	579 (23.3)	553 (22)	516 (21.1)	635 (24.7)	
High school or equivalent	2237 (22.3)	497 (20)	530 (21.1)	577 (23.6)	633 (24.7)	
College or above	5489 (54.8)	1413 (56.8)	1430 (56.9)	1348 (55.2)	1298 (50.6)	
Marital status, n (%)						<.001
Married	5086 (50.8)	1198 (48.1)	1285 (51.1)	1259 (51.6)	1344 (52.4)	
Widowed	979 (9.8)	200 (8)	187 (7.4)	207 (8.5)	385 (15)	
Divorced	1029 (10.3)	266 (10.7)	224 (8.9)	259 (10.6)	280 (10.9)	
Separated	328 (3.3)	74 (3)	108 (4.3)	73 (3)	73 (2.8)	
Never married	1790 (17.9)	513 (20.6)	491 (19.5)	448 (18.4)	338 (13.2)	
Living with partner	797 (8.0)	238 (9.6)	218 (8.7)	195 (8)	146 (5.7)	
PIR, median (IQR)	2.3 (1.2–3.7)	2.2 (1.1–3.5)	2.4 (1.1–3.8)	2.4 (1.2–3.9)	2.4 (1.3–3.6)	.008
Hypertension, n (%)						<.001
No	5451 (54.5)	1358 (54.6)	1538 (61.2)	1418 (58.1)	1137 (44.3)	
Yes	4558 (45.5)	1131 (45.4)	975 (38.8)	1023 (41.9)	1429 (55.7)	
High cholesterol level, n (%)						<.001
No	6434 (64.3)	1735 (69.7)	1705 (67.8)	1558 (63.8)	1436 (56)	
Yes	3575 (35.7)	754 (30.3)	808 (32.2)	883 (36.2)	1130 (44)	
Diabetes, n (%)						<.001
No	8576 (85.7)	2250 (90.4)	2222 (88.4)	2126 (87.1)	1978 (77.1)	
Yes	1433 (14.3)	239 (9.6)	291 (11.6)	315 (12.9)	588 (22.9)	
Depression, n (%)						.928
No	9274 (92.7)	2299 (92.4)	2343 (93.2)	2256 (92.4)	2376 (92.6)	
Yes	735 (7.3)	190 (7.6)	170 (6.8)	185 (7.6)	190 (7.4)	
Sleep disorder, n (%)						.302
No	7391 (73.8)	1877 (75.4)	1895 (75.4)	1755 (71.9)	1864 (72.6)	
Yes	2618 (26.2)	612 (24.6)	618 (24.6)	686 (28.1)	702 (27.4)	
BMI (kg/m^2^), median (IQR)	28.1 (24.4–32.6)	27.6 (23.7–32.0)	28.0 (24.3–32.5)	28.5 (24.7–33.0)	28.4 (24.7–32.8)	<.001
Serum creatinine (mg/dL), median (IQR)	0.9 (0.7–1.0)	0.8 (0.7–1.0)	0.8 (0.7–1.0)	0.8 (0.7–1.0)	0.9 (0.8–1.1)	<.001
Serum total calcium (mg/dL), median (IQR)	9.4 (9.1–9.6)	9.3 (9.1–9.6)	9.3 (9.1–9.6)	9.4 (9.2–9.6)	9.4 (9.2–9.6)	<.001
Serum sodium (mmol/L), median (IQR)	139.0 (138.0–140.0)	139.0 (138.0–140.0)	139.0 (138.0–140.0)	139.0 (138.0–140.0)	139.0 (138.0–141.0)	.035
Daily potassium intake (mg), median (IQR)	2541.0 (1894.0–3127.0)	2397.0 (1741.0–2955.0)	2489.0 (1880.0–3088.0)	2594.0 (1956.0–3274.0)	2605.6 (1986.2–3173.5)	<.001
Daily alcohol intake (g), median (IQR)	0.0 (0.0–8.0)	0.0 (0.0–7.0)	0.0 (0.0–8.0)	0.0 (0.0–8.0)	0.0 (0.0–8.0)	<.001
Smoking status, n (%)						<.001
Never	5628 (56.2)	1539 (61.8)	1508 (60)	1331 (54.5)	1250 (48.7)	
Former	2571 (25.7)	500 (20.1)	564 (22.4)	648 (26.5)	859 (33.5)	
Current	1810 (18.1)	450 (18.1)	441 (17.5)	462 (18.9)	457 (17.8)	
Ever exposure to firearm noise, n (%)						<.001
No	6130 (61.2)	1687 (67.8)	1624 (64.6)	1426 (58.4)	1393 (54.3)	
Yes	3879 (38.8)	802 (32.2)	889 (35.4)	1015 (41.6)	1173 (45.7)	
Ever exposure to loud noise, n (%)						<.001
No	7917 (79.1)	2032 (81.6)	2063 (82.1)	1987 (81.4)	1835 (71.5)	
Yes	2092 (20.9)	457 (18.4)	450 (17.9)	454 (18.6)	731 (28.5)	

Statistical comparisons were performed using weighted chi-square tests for categorical variables and weighted Kruskal–Wallis tests for continuous variables. *P* values <.05 were considered statistically significant.

BMI = body mass index, HFHL = high-frequency hearing loss, IQR = interquartile range, LFHL = low-frequency hearing loss, PIR = poverty income ratio, SFHL = speech-frequency hearing loss.

### 3.3. The association between serum potassium levels and the prevalence of hearing loss

**Figure 3.**
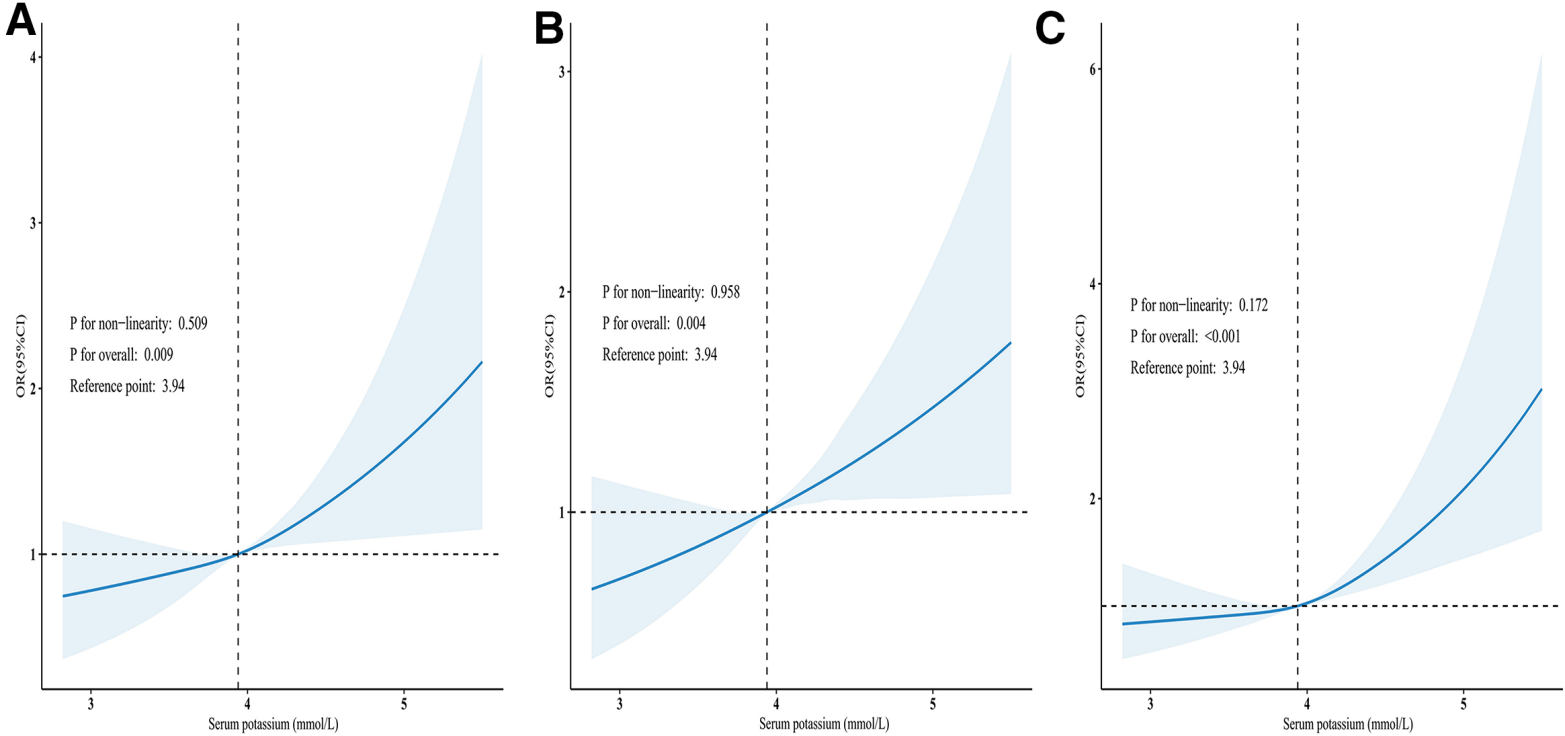
RCS analysis with multivariate-adjusted associations between serum potassium levels and hearing loss. (A) SFHL, (B) LFHL, and (C) HFHL. The reference point for serum potassium is 3.94 mmol/L. CI = confidence interval, HFHL = high-frequency hearing loss, LFHL = low-frequency hearing loss, OR = odds ratio, RCS = restricted cubic spline, SFHL = speech-frequency hearing loss.

After multivariable adjustment (model 3), serum potassium levels (as a continuous variable) were significantly positively associated with the prevalence of hearing loss. For each 1 mmol/L increase in serum potassium level, the odds of SFHL increased by 49% (OR = 1.49, 95% CI: 1.15–1.94, *P* = .003), the prevalence of LFHL increased by 45% (OR = 1.45, 95% CI: 1.16–1.82, *P* = .002), and the prevalence of HFHL increased by 57% (OR = 1.57, 95% CI: 1.29–1.92, *P* < .001) (Table 2). Based on the RCS analysis, serum potassium levels were positively associated with the prevalence of all 3 types of hearing loss. Using the median value of 3.94 mmol/L as the reference point, SFHL (*P* for overall = .009, *P* for nonlinearity = .509), LFHL (*P* for overall = .004, *P* for nonlinearity = .958), and HFHL (*P* for overall <.001, *P* for nonlinearity = .172) all showed statistically significant overall trends, but no evidence of nonlinearity was observed (Fig. 3). These results suggest that higher serum potassium levels are associated with a higher prevalence of hearing loss, particularly in higher frequency ranges, highlighting the importance of serum potassium as a potential risk factor for hearing loss.

**Table 2:** Multivariate logistic regression models used to analyze the associations between serum potassium levels and the prevalence of hearing loss.

		Model 1		Model 2		Model 3	
Outcomes	Exposure	OR (95% CI)	*P* value	OR (95% CI)	*P* value	OR (95% CI)	*P* value
SFHL	Continuous	3.77 (3.04–4.67)	<.001	1.57 (1.25–1.97)	<.001	1.49 (1.15–1.94)	.003
	Quartile						
	*Q*1	Reference		Reference		Reference	
	*Q*2	1.05 (0.86–1.28)	.622	0.94 (0.71–1.25)	.681	0.96 (0.73–1.28)	.800
	*Q*3	1.44 (1.18–1.75)	<.001	1.19 (0.96–1.47)	.103	1.24 (0.99–1.56)	.061
	*Q*4	3.08 (2.54–3.73)	<.001	1.49 (1.20–1.84)	<.001	1.45 (1.16–1.81)	.002
	*P* for trend		<.001		<.001		<.001
LFHL	Continuous	3.28 (2.72–3.96)	<.001	1.51 (1.24–1.85)	<.001	1.45 (1.16–1.82)	.002
	Quartile						
	*Q*1	Reference		Reference		Reference	
	*Q*2	1.03 (0.84–1.26)	.757	0.97 (0.76–1.23)	.779	0.98 (0.77–1.26)	.879
	*Q*3	1.36 (1.10–1.70)	.006	1.20 (0.96–1.49)	.106	1.25 (0.99–1.57)	.058
	*Q*4	2.82 (2.39–3.33)	<.001	1.52 (1.26–1.84)	<.001	1.50 (1.22–1.85)	<.001
	*P* for trend		<.001		<.001		<.001
HFHL	Continuous	3.78 (3.11–4.59)	<.001	1.65 (1.36–2.00)	<.001	1.57 (1.29–1.92)	<.001
	Quartile						
	*Q*1	Reference		Reference		Reference	
	*Q*2	1.16 (0.98–1.38)	.088	1.08 (0.88–1.32)	.472	1.09 (0.89–1.35)	.395
	*Q*3	1.49 (1.26–1.76)	<.001	1.19 (0.96–1.47)	.111	1.20 (0.97–1.49)	.098
	*Q*4	3.15 (2.56–3.87)	<.001	1.50 (1.20–1.87)	<.001	1.44 (1.15–1.80)	.002
	*P* for trend		<.001		<.001		.001

Model 1: Unadjusted model.

Model 2: Adjusted for age, sex, race/ethnicity, education level, marital status, and PIR.

Model 3: Additionally adjusted for hypertension, high cholesterol, diabetes, depression, sleep disorder, BMI, serum creatinine, serum total calcium, serum sodium, daily potassium intake, daily alcohol intake, smoking status, and exposure to firearm or loud noise.

CI = confidence interval, HFHL = high-frequency hearing loss, LFHL = low-frequency hearing loss, OR = odds ratio, SFHL = speech-frequency hearing loss.

### 3.4. Subgroup analyses and interaction effects

**Figure 4.**
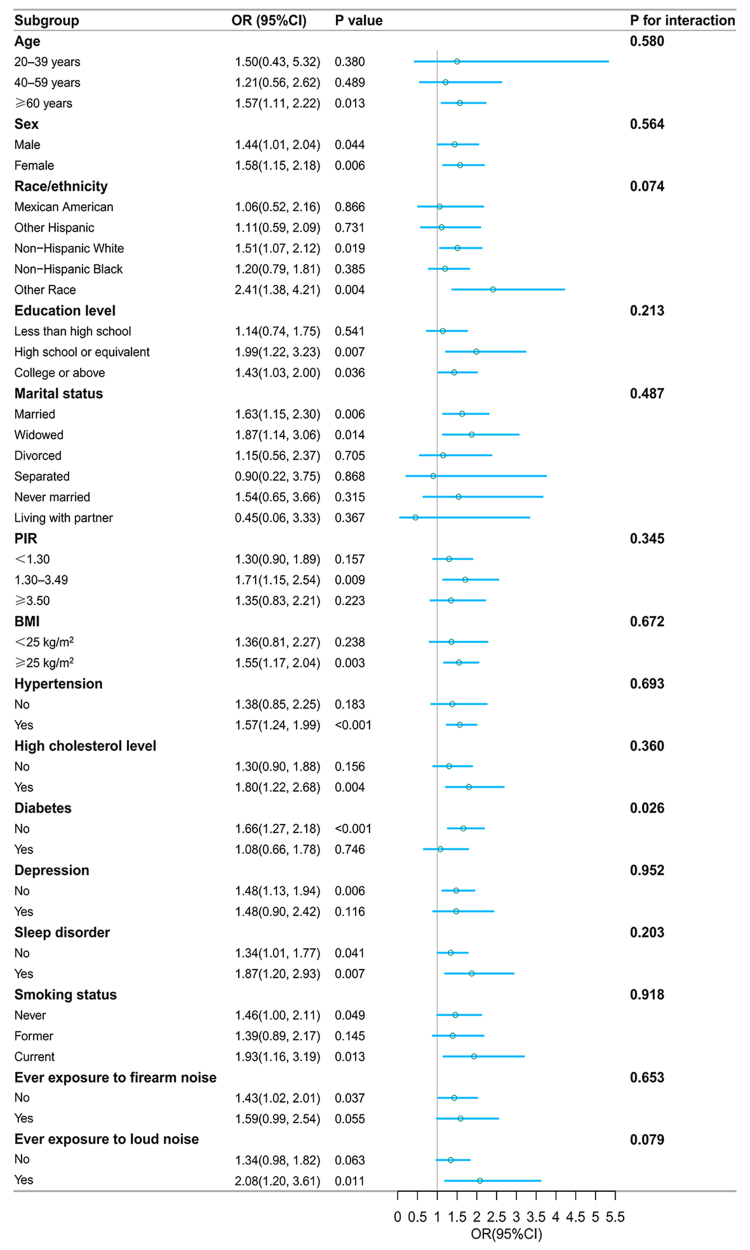
Subgroup analysis of the association between serum potassium levels and SFHL. BMI = body mass index, CI = confidence interval, OR = odds ratio, PIR = poverty income ratio, SFHL = speech-frequency hearing loss.

**Figure 5.**
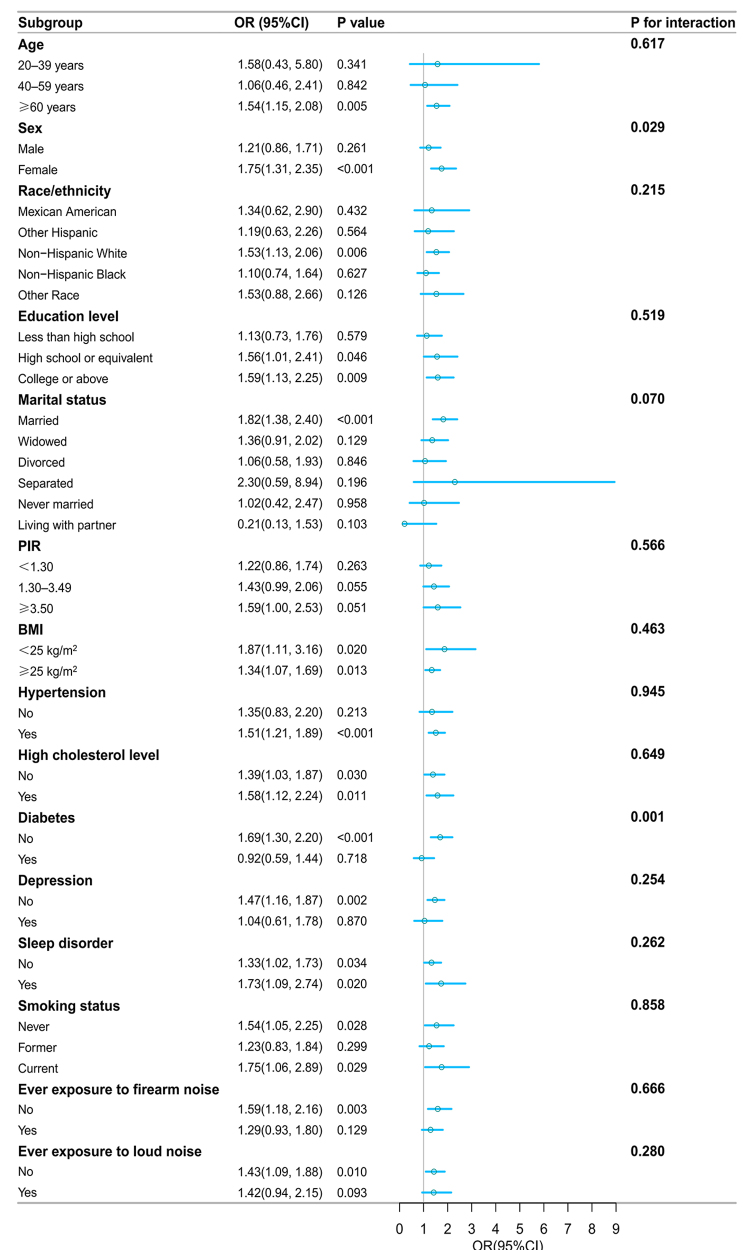
Subgroup analysis of the association between serum potassium levels and LFHL. BMI = body mass index, CI = confidence interval, LFHL = low-frequency hearing loss, OR = odds ratio, PIR = poverty income ratio.

**Figure 6.**
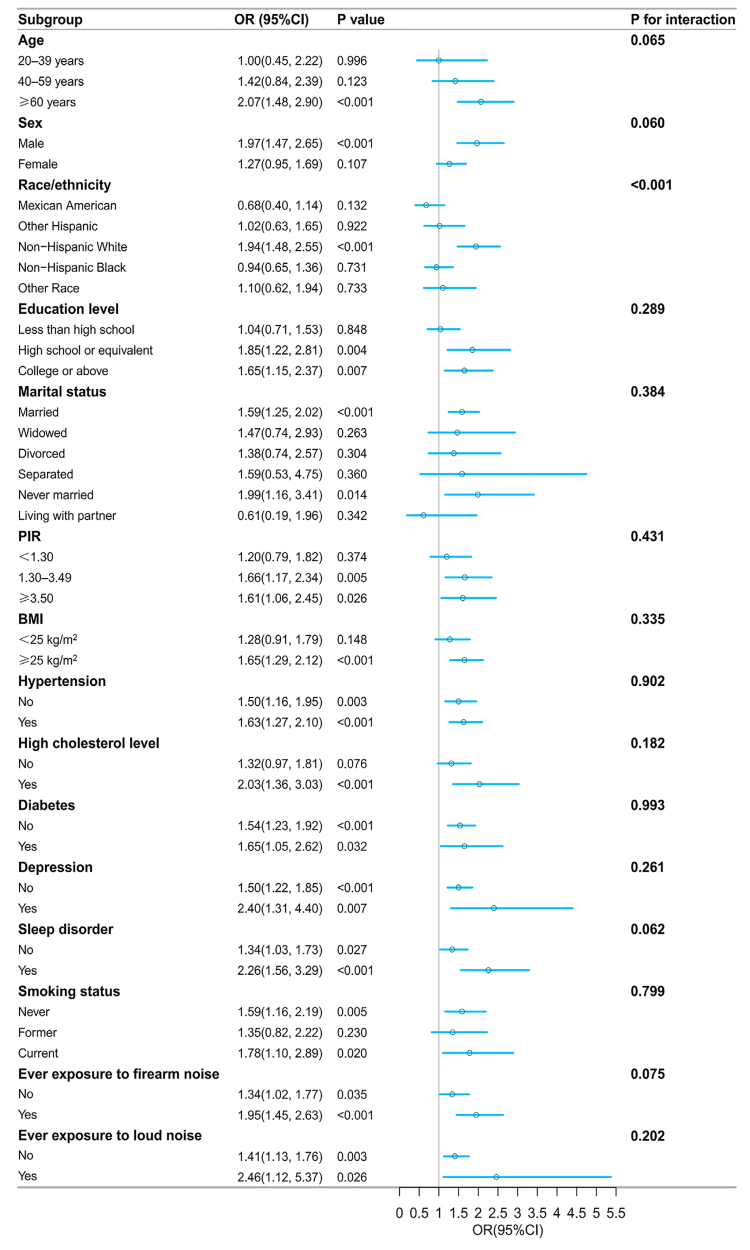
Subgroup analysis of the association between serum potassium levels and HFHL. BMI = body mass index, CI = confidence interval, HFHL = high-frequency hearing loss, OR = odds ratio, PIR = poverty income ratio.

In the subgroup analyses, serum potassium levels were positively associated with the prevalence of SFHL, LFHL, and HFHL across multiple subgroups. Interaction analyses revealed a significant interaction between diabetes status and serum potassium levels in relation to SFHL (*P* for interaction = .026), indicating that the association between serum potassium and hearing loss was more pronounced among participants without diabetes (Fig. 4). For LFHL, significant interactions were observed for sex (*P* for interaction = .029) and diabetes status (*P* for interaction = .001), suggesting that the positive association between serum potassium levels and LFHL was stronger in females and participants without diabetes (Fig. 5). Race/ethnicity showed a significant interaction effect in the association between serum potassium levels and HFHL (*P* for interaction <.001), with the strongest and most significant association observed among non-Hispanic White participants (Fig. 6).

## 4. Discussion

Hearing loss, associated with various metabolic factors, has emerged as a significant global health issue.^[18,19]^ This study utilized data from the NHANES database to explore the association between serum potassium levels and hearing loss. The results indicated that higher serum potassium levels were significantly positively associated with hearing loss, and this association remained significant even after adjusting for multiple covariates. RCS analysis revealed a significant positive linear association between serum potassium levels and SFHL, LFHL, and HFHL. These findings suggest that serum potassium levels may play a crucial role in the development of hearing loss, with distinct patterns of association observed across different frequency ranges.

Previous studies have suggested that potassium intake may play an important role in maintaining auditory function. Jung et al investigated the association between potassium intake and hearing thresholds among Korean adults using data from the Korean National Health and Nutrition Examination Survey. Participants were divided into tertiles based on potassium intake: low (0–1338 mg/1000 kcal), medium (1339–1761 mg/1000 kcal), and high (1762–10,384 mg/1000 kcal), with 1975 individuals in each group. Pure-tone audiometry tests were conducted to calculate the average thresholds for low, mid, and high frequencies, as well as the overall average hearing threshold. Hearing loss was defined as an average hearing threshold of the better ear exceeding 40 dB. The results showed that as potassium intake increased, all 4 average hearing thresholds significantly decreased. After adjusting for various covariates, including age, diabetes, hypertension, household income, smoking status, alcohol consumption, educational level, physical activity, estimated glomerular filtration rate, calorie intake, protein intake, fat intake, carbohydrate intake, sodium intake, potassium intake, occupational noise exposure, and explosive noise exposure, multivariable analyses revealed that the hearing thresholds in the high-potassium group were significantly lower than those in other groups. Additionally, potassium intake was negatively correlated with hearing thresholds. These findings suggest that high potassium intake is associated with a lower prevalence of hearing loss and better hearing thresholds (i.e., better hearing ability).^[20]^

In contrast to the findings of Jung et al, our study revealed that individuals with higher serum potassium levels were associated with a higher prevalence of hearing loss. Our study differs from previous research in the following aspects. First, the study population consisted of Americans, whereas Jung et al focused on a Korean population. Differences in geography and ethnicity may affect the comparability of the results. Second, unlike Jung et al, who used estimated dietary potassium intake, our study directly measured serum potassium levels. Dietary potassium intake and serum potassium levels do not necessarily represent the same biological construct because circulating potassium is tightly regulated by renal excretion, hormonal control, acid–base balance, and transcellular shifts.^[21]^ Therefore, higher serum potassium levels, even after adjustment for serum creatinine, may reflect altered potassium handling or cellular redistribution rather than simple dietary overconsumption. Third, Jung et al defined hearing loss as PTA ≥ 40 dB, whereas our study used a definition based on a PTA ≥ 20 dB, potentially leading to differences in the classification of hearing loss. Fourth, our study included a larger sample size of 10,009 participants, covering a more representative population. Fifth, a broader range of covariates was adjusted for to minimize the influence of potential confounding factors. Sixth, RCS analysis was employed to explore the nonlinear association between serum potassium levels and hearing loss, while subgroup and interaction analyses were conducted to assess the stability and variability of this association across different populations. These methodological improvements enhance the scientific rigor and robustness of our study, making the findings more compelling and reliable.

The following inner-ear-related mechanisms should be interpreted as plausible explanations only, because hearing loss was defined using air-conduction audiometry and could not be definitively classified as sensorineural or conductive despite the use of otoscopy and tympanometry in the NHANES protocol. High serum potassium may affect hearing through several mechanisms. First, elevated potassium levels may disrupt the ion gradient in the inner ear, affecting the electrical potential changes in hair cells, thereby leading to a decline in auditory function. Abnormally high potassium concentrations may induce an excessively positive endocochlear potential, negatively impacting hair cell function.^[22–25]^ Second, higher serum potassium levels may cause excessive depolarization of cell membranes, interfering with neural conduction and synaptic activity. These changes may result in abnormal signal processing in the inner ear and central auditory pathways, ultimately leading to hearing loss.^[9,26–28]^ Third, potassium imbalance may cause abnormalities in cochlear blood vessels, such as vasoconstriction or vasodilation, altering the microcirculation in the inner ear. This may interfere with the nutrient supply to hair cells and the maintenance of their function.^[29–31]^ Fourth, elevated potassium levels may exacerbate genetic disorders related to potassium channels. For example, mutations in the KCNQ4 gene can impair potassium channel function in the inner ear, leading to hair cell dysfunction and, ultimately, hearing loss.^[32–35]^ Fifth, in some cases, elevated serum potassium levels may lead to imbalances in other ions, such as sodium and calcium, affecting ion exchange between hair cells and supporting cells, further aggravating hearing impairment.^[11,22,36]^ Sixth, higher serum potassium levels may be associated with other metabolic disorders, such as chronic kidney disease or endocrine dysfunction. These metabolic abnormalities may jointly contribute to or worsen hearing impairment.^[37–40]^ In addition, potassium imbalance and sodium restriction may be relevant to inner-ear fluid and ionic homeostasis. A salt-restricted diet has been described as part of lifestyle counseling for Ménière disease,^[41]^ while ion transport in the endolymphatic sac and K^+^ channel activity may be involved in maintaining the ionic milieu of inner-ear fluid.^[42]^ This concept is mechanistically connected with potassium channel dysfunction, such as KCNQ4-related abnormalities, and alterations in cochlear microcirculation, both of which may contribute to auditory dysfunction.^[29–35]^ However, further prospective studies are needed to better explore the complex association between serum potassium levels and hearing loss.

## 5. Limitations

Several limitations of this study should be noted. First, cross-sectional design and causality: As this study is based on cross-sectional data, we cannot establish a causal relationship between serum potassium levels and hearing loss. The findings are limited to an exploration of their association. Future large-scale longitudinal studies are needed to verify the causal nature of this relationship. Second, limitations of data source: the data used in this study were derived from the NHANES database, which, while nationally representative, is based on a specific sample of the U.S. population. Consequently, the findings may not be generalizable to other racial or regional populations. Third, potential confounding factors: Although we adjusted for numerous covariates that might influence serum potassium levels and hearing loss, some unmeasured confounding factors may still exist, potentially affecting the results. Fourth, the definition of hearing loss in this study was based on a PTA threshold of ≥20 dB. Other studies may use different criteria for hearing loss, limiting the comparability of findings across studies. Fifth, we relied on a single measurement of serum potassium levels, which may reflect short-term physiological, dietary, or hydration-related fluctuations rather than long-term potassium homeostasis. Sixth, hearing loss was defined using air-conductionpure-tone audiometry, and differentiation between sensorineural and conductive hearing loss was not possible. These limitations highlight the need for further research to address these challenges and strengthen the understanding of the association between serum potassium levels and hearing loss.

## 6. Conclusion

Our findings reveal a notable positive association between serum potassium levels and the prevalence of hearing loss. Elevated serum potassium levels may be associated with a higher prevalence of hearing loss through mechanisms such as altering the electrical activity of cochlear hair cells and disrupting potassium ion balance in the inner ear. These findings contribute to a deeper understanding of the role of metabolic factors in auditory health and provide new directions for future research on the prevention and intervention of hearing loss. In light of these findings, it is crucial to emphasize the importance of regular laboratory testing in cases of hearing loss to monitor metabolic disturbances, such as potassium imbalances. Dietary changes, particularly regulating potassium intake and restricting sodium, could play a significant role in managing inner ear disorders and preventing further auditory damage. However, further longitudinal studies are needed to validate this association and elucidate its underlying mechanisms.

## Author contributions

**Conceptualization:** Xiao-Zou Luo, Jian Luo.

**Data curation:** Xiao-Zou Luo, Jian Luo, Jie Shen, Deng-Chao Wang.

**Investigation:** Jie Shen, Deng-Chao Wang.

**Methodology:** Deng-Chao Wang.

**Supervision:** Xiao-Zou Luo.

**Visualization:** Jie Shen.

**Writing – original draft:** Xiao-Zou Luo, Jian Luo, Jie Shen, Deng-Chao Wang.

**Writing – review & editing:** Xiao-Zou Luo, Jian Luo, Jie Shen, Deng-Chao Wang.
